# IL23 and TGF-ß diminish macrophage associated metastasis in pancreatic carcinoma

**DOI:** 10.1038/s41598-018-24194-5

**Published:** 2018-04-11

**Authors:** S. Mazher Hussain, Leighton F. Reed, Bradley A. Krasnick, Gustavo Miranda-Carboni, Ryan C. Fields, Ye Bi, Abul Elahi, Abidemi Ajidahun, Paxton V. Dickson, Jeremiah L. Deneve, William G. Hawkins, David Shibata, Evan S. Glazer

**Affiliations:** 1 University of Tennessee Health Science Center, Memphis, TN USA; 2 Barnes-Jewish Hospital and The Alvin J. Siteman Cancer Center, Washington University in St. Louis, St. Louis, MO USA; 3 UT West Cancer Center, Memphis, TN USA

## Abstract

The precise role of tumor associated macrophages remains unclear in pancreatic ductal adenocarcinoma (PDAC) while TGF-ß has an unclear role in metastases formation. In order to understand the role of IL23, an interleukin associated with macrophage polarization, we investigated IL23 in the context of TGF-ß expression in PDAC. We hypothesized that IL23 expression is associated with metastatic development and survival in PDAC. We investigated IL23 and TGF-ß protein expression on resected PDAC patient tumor sections who were divided into short-term (<12 months) survivors and long-term (>30 months) survivors. Panc-1 cells treated with IL23, TGF-ß, macrophages, or combinations thereof, were orthotopically implanted into NSG mice. Patients in the long-term survivor group had higher IL23 protein expression (P = 0.01). IL23 expression was linearly correlated with TGF-ß expression in patients in the short-term survivor group (P = 0.038). Macrophages induce a higher rate of PDAC metastasis in the mouse model (P = 0.02), which is abrogated by IL23 and TGF-ß treatment (P < 0.001). Macrophages serve a critical role in PDAC tumor growth and metastasis. TGF-ß contributes to a less tumorigenic TME through regulation of macrophages. Macrophages increases PDAC primary tumor growth and metastases formation while combined IL23 and TGF-ß pre-treatment diminishes these processes.

## Introduction

Pancreatic ductal adenocarcinoma (PDAC) carries one of the highest case fatality rate of any cancer and will likely become the second leading cause of cancer related death in the next decade due to a rising incidence^1,2^. PDAC tumorigenesis is estimated to take approximately 10 years with a series of molecular and genetic events forming the malignancy from pre-malignant lesions^3^. While the identification of 4 subtypes of PDAC has clarified tumorigenesis, and with chronic inflammation serving as a risk factor, the role of inflammation in malignant progression remains unclear^4,5^. Growing evidence demonstrates the importance of macrophages in driving peri-tumoral inflammation during malignant progression^5,6^.

Targeting inflammation in PDAC is a reasoned approach based on data demonstrating intra-tumoral and whole body inflammation in patients with PDAC^7,8^. While there is clear inflammation, fibrosis, and immunogenicity in the pancreatic tumor microenvironment (TME), harnessing the immune system to treat PDAC has remained elusive^6,9,10^. There is a growing evidence that tumor associated macrophages define a novel type of macrophages with mixed polarization (referred to as macrophage M1 and M2 polarization) that describe the immunosuppressive TME with concordant inflammation and fibrosis^5,11–14^.

TGF-ß has a paradoxical relationship with survival in PDAC patients where it is a tumor suppressor in early stage PDAC and a tumor promotor in late stage PDAC^15–17^. While TGF-ß is known to drive inflammation in the TME, the role of interleukins in this TGF-ß paradox is not well understood^16,18^. Recent work has demonstrated the interaction between Smad4, a downstream mediator of TGF-ß, and Sox4, a transcription factor critical for embryologic development, partially determines whether TGF-ß will act as a tumor suppressor or tumor promoter^16^. A limitation of this model is the lack of interaction with the TME in advanced PDAC tumors, where the amount of TGF-ß expression is known to correlate with overall tumor burden and suggests the amount of protein expression may partially explain this paradox^19^.

Interluekin-23 (IL23) is a pro-inflammatory protein that is part of the IL12 family of proteins and believed to serve a critical role in the TME of several malignancies^20–22^. It is secreted by tumor associated macrophages, and it has been suggested that IL23 and TGF-ß both modulate inflammation in the TME^5,6^. In esophageal cancer, IL23 promotes epithelial to mesenchymal transition (EMT) and is associated with overall poor prognosis^20,21^. Other data has suggested that IL23 actually provides an immune mediated protective effect against some sarcomas and carcinomas via inducing apoptosis in malignant cells^23–25^. IL23 may have distinct roles in primary tumor growth and metastasis based on the model or disease site under investigation^26–28^. In PDAC patients, serum IL23 levels have been shown to be higher in metastatic patients but it is unclear if this is related to TGF-ß expression^28^. In contrast to this, more recent data has shown low IL23 expression in metastatic PDAC patients in the setting of high TGF-ß expression^29^.

In order to better define the role of IL23 in PDAC biology, we investigated it in the context of TGF-ß expression. An underlying theory is that anti-TGF-ß based therapy will only be effective in patients with certain but not all highly inflammatory TME. We hypothesized that IL23 expression is associated with survival in PDAC. We evaluated on the interaction between macrophages and PDAC cells through IL23 and TGF-ß cross talk.

## Results

### Patients

Ten of the 24 patients in the short-term survivor group were male (44%) while 11 of 24 in the long-term survivor group were male (46%). The average age ± standard deviation of patients in the short-term survivor group (65.8 ± 12.0 years) was similar to the average age in the long-term survivor group (66.2 ± 9.0 years, P = 0.9). All patients were stage I or II. Seventy-five percent of the patients in the short-term survivor group had tumor lymphovascular invasion (LVI) while only 50% of patients in the long-term survivor group had tumors with LVI (P = 0.044).

### Expression of IL23 and TGF-ß in resected specimens

**Figure 1:**
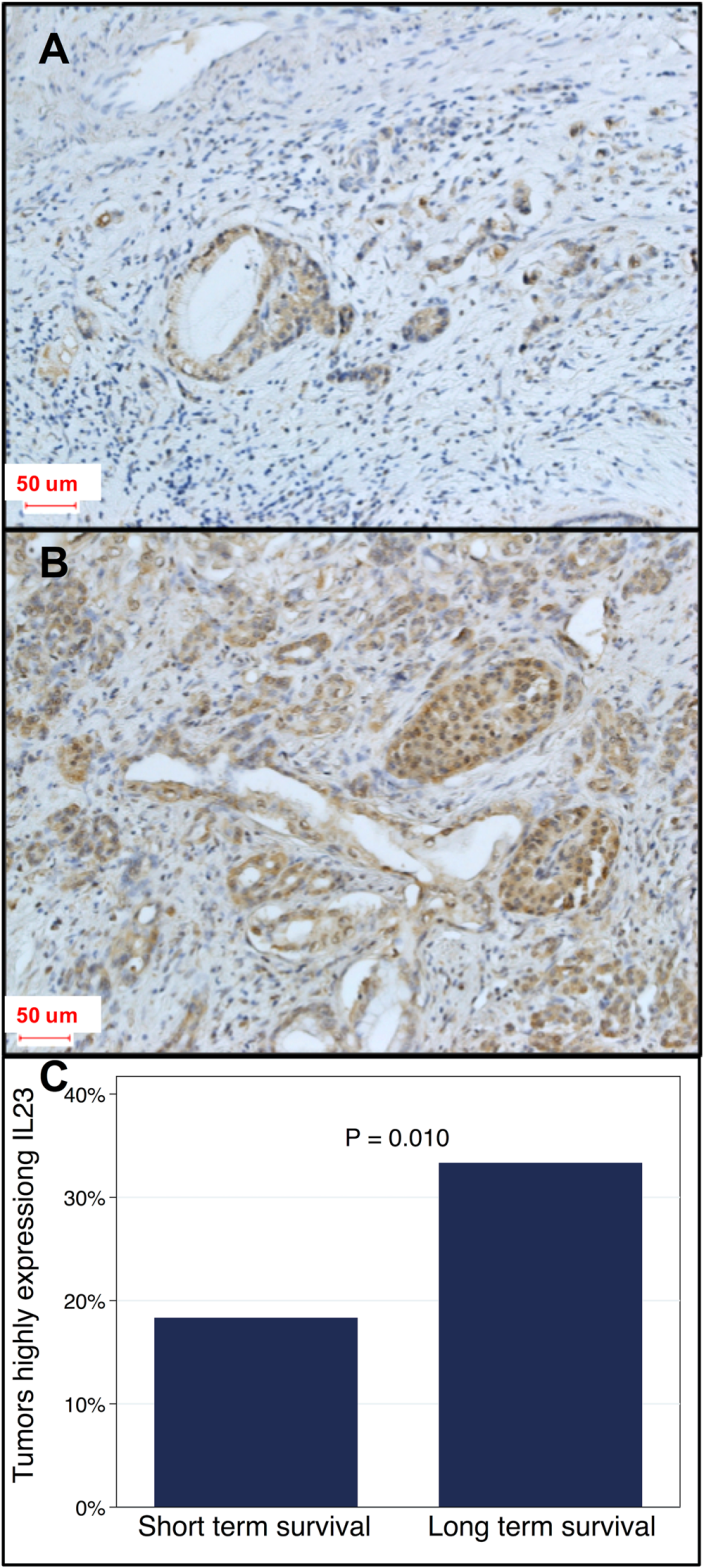
IL23 protein expression in patient tumor specimens. Immunohistochemistry of IL23 from tumors in patients with short-term survivors (**A**) were more likely to have low IL23 expression compared to long-term survivors which had higher IL23 expression (**B**, OR = 0.48, P = 0.019). Quantification of IL23 from patients in panel A and B. Tumors from long-term survivors were more likely to highly express IL23 (**C**, P = 0.010).

In order to investigate the relationships between IL23 and TGF-ß, we investigated the correlation between IL23 and TGF-ß protein expression and survival status utilizing immunohistochemistry (IHC). We compared tumors in both short-term and long-term patient survivorships (Fig. 1). The quartile ranges for protein expression were based on quantitative protein expression analysis of all tumor specimens combined (both long- and short-term groups combine to define the 25^th^ and 75^th^ percentiles defining the upper quartile, the lower quartile, and the inter-quartile range for each protein).

**Figure 2:**
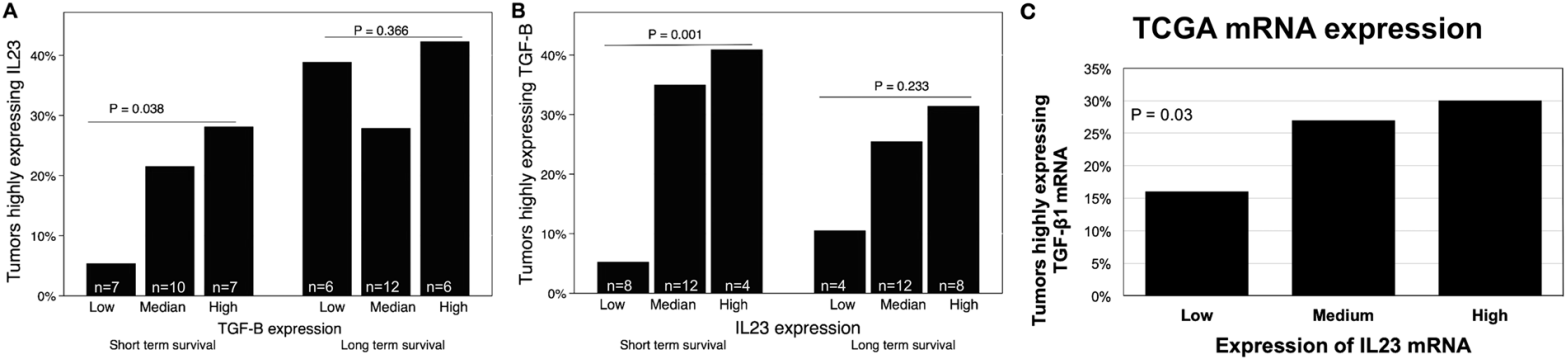
Relationship between IL23 and TGF-ß protein expression in patient tumor specimens. For short-term survivors, IL23 tumor expression is linearly correlated with TGF-ß tumor expression (P < 0.05, left side of **A**,**B**) whereas IL23 expression is not correlated with TGF-ß expression in long-term survivors (P > 0.2, right side, **A** and **B**). Data from the TCGA demonstrated correlation between IL23 and TGF-ß1 gene expression in those PDAC tumor specimens (P = 0.03, **C**).

The data demonstrates IL23 protein expression is found to be positive (equivalent to a 3+ score) in the patients who survive for long-term and relatively negative (equivalent to a 0/1+ score) in the short-term patients. Histomorphometric quantification analysis suggests that high IL23 protein expression is statically associated with survivorship (Fig. 1C). We found that IHC protein expression of IL23 and TGF-ß is highly correlated in short term (Fig. 2, P < 0.001) but not in long-term (P = 0.10) survivors. Overall, tumors from short-term survivors demonstrated lower mean TGF-ß expression (OR = 0.46, P = 0.017) and lower mean IL23 expression (OR = 0.48, P = 0.019) compared to tumors from long term survivors.

Upon separating protein expression into high (highest quartile), low (lowest quartile) and median (interquartile range) expression levels, tumors from long term survivors were associated with 20% higher IL23 protein expression than tumors from short-term survivors (P = 0.008; data not shown). For short-term survivors, IL23 tumor expression is linearly correlated with TGF-ß tumor expression (P < 0.05, left side of Fig. 2A,B). IL23 expression is not correlated with TGF-ß expression in long-term survivors (P > 0.2, right side of Fig. 2A,B). There was no association between IL23 protein expression and the survival group when TGF-ß protein expression was in the low or median range (P > 0.5). Multivariate analysis demonstrated that long term survival was linearly associated with increasing IL23 expression (OR = 3, P = 0.001) but not TGF-ß expression (P = 0.07). We also found that IL23 expression and TGF-ß expression in long-term survivors was statistically correlated (P = 0.037), suggesting a reason that each protein expression was not associated with survival in isolation.

### IL23 and TGF-ß have an integral relationship in the pancreatic TME

Since protein expression of IL23 and TGF-ß were significantly correlated in short term surviving patients (Fig. 2A,B), we sought to understand if there was a relationship level at the gene expression level. In order to understand the relationship between IL23 and TGF-ß gene expression in PDAC cancer cells, we utilized The Cancer Genome Atlas (TCGA) data set to investigate the association between the gene products for *IL23* and *TGF-ß1*. In this readily accessible, high quality, and validated data set, we found that the proportion of tumors with highly expressing *TGF-ß1* gene expression is linearly associated with *IL23* gene expression by quartile (Fig. 2C, P = 0.03).

### Macrophages transiently express IL23

**Figure 3:**
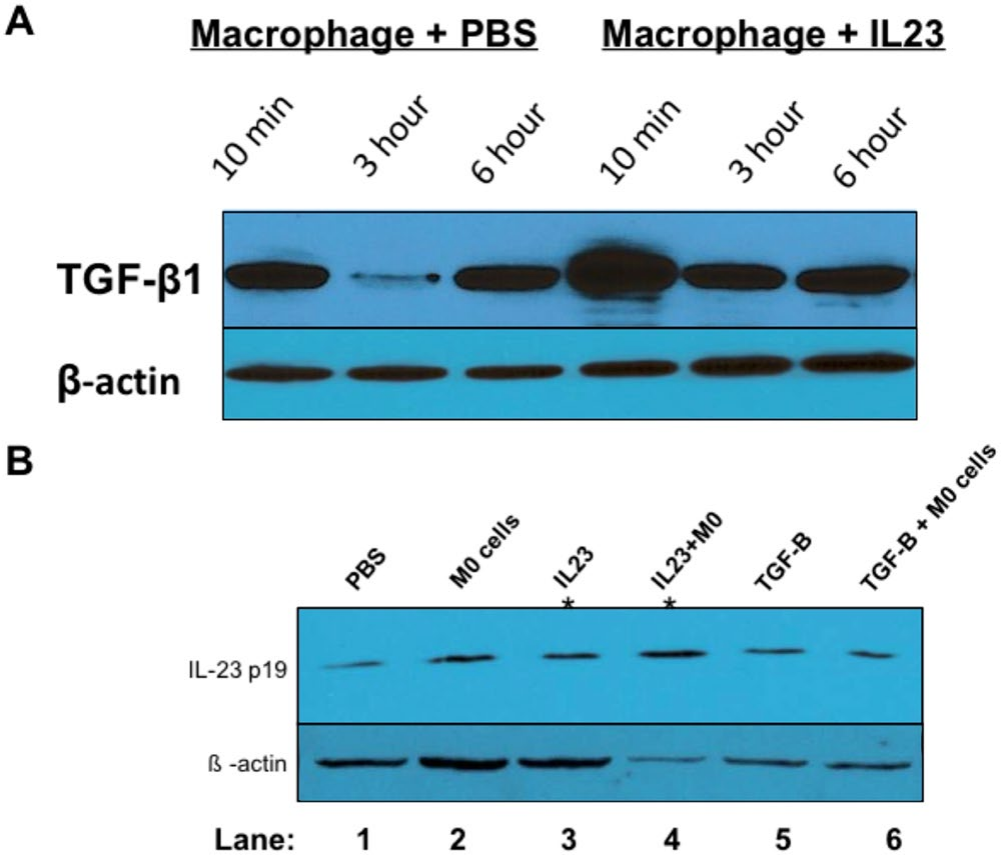
Relationship between IL23 and TGF-ß protein expression *in vitro*. We found that IL23 transiently increases TGF-ß1 expression in a macrophage cell line (**A**). IL23 is slightly increased in Panc-1 cells after macrophage exposure, TGF-ß treatment, and TGF-ß + macrophage treatment (Lanes 2, 5, and 6 in B, respectively) compared to PBS treated Panc-1 cells (Lane 1) and IL23 treated Panc-1 cells (lanes 3 & 4, positive controls marked *).

We then investigated the expression of IL23 *in vitro* through an activated macrophage cell line (ATCC 9855). We partially polarized this cell line towards the M2 phenotype in order to recapitulate the tumor associated macrophage phenotype in this model^11^. We found that IL23 transiently increases TGF-ß1 expression in this macrophage cell line after 10 minutes of IL23 treatment, and it is diminished by 3 hours of IL23 treatment (Fig. 3A). At 3 hours of PBS treatment, macrophages demonstrate a temporary decrease in TGF-ß1 production similar to other studies demonstrating macrophage response (decreased growth factors) to saline^30,31^. We did not find that IL23 or TGF-ß1 expression in macrophages *in vitro* altered nitric oxide synthase expression (data not shown).

In investigating the effects of IL23 on Panc-1 cells exposed to macrophages, we found that IL23 produced by Panc-1 cells is slightly increased in after TGF-ß treatment (Fig. 3B) when compared to PBS controls. Positive controls with IL23 added (Fig. 2B, Lanes 3 & 4). The increased expression of IL23 was most seen in the presences of macrophages with TGF-ß treatment slightly modulating that expression (Fig. 3B).

### IL23 and TGF-ß decrease the macrophage induced metastatic potential of PDAC cells when implanted into a mouse model

**Figure 4:**
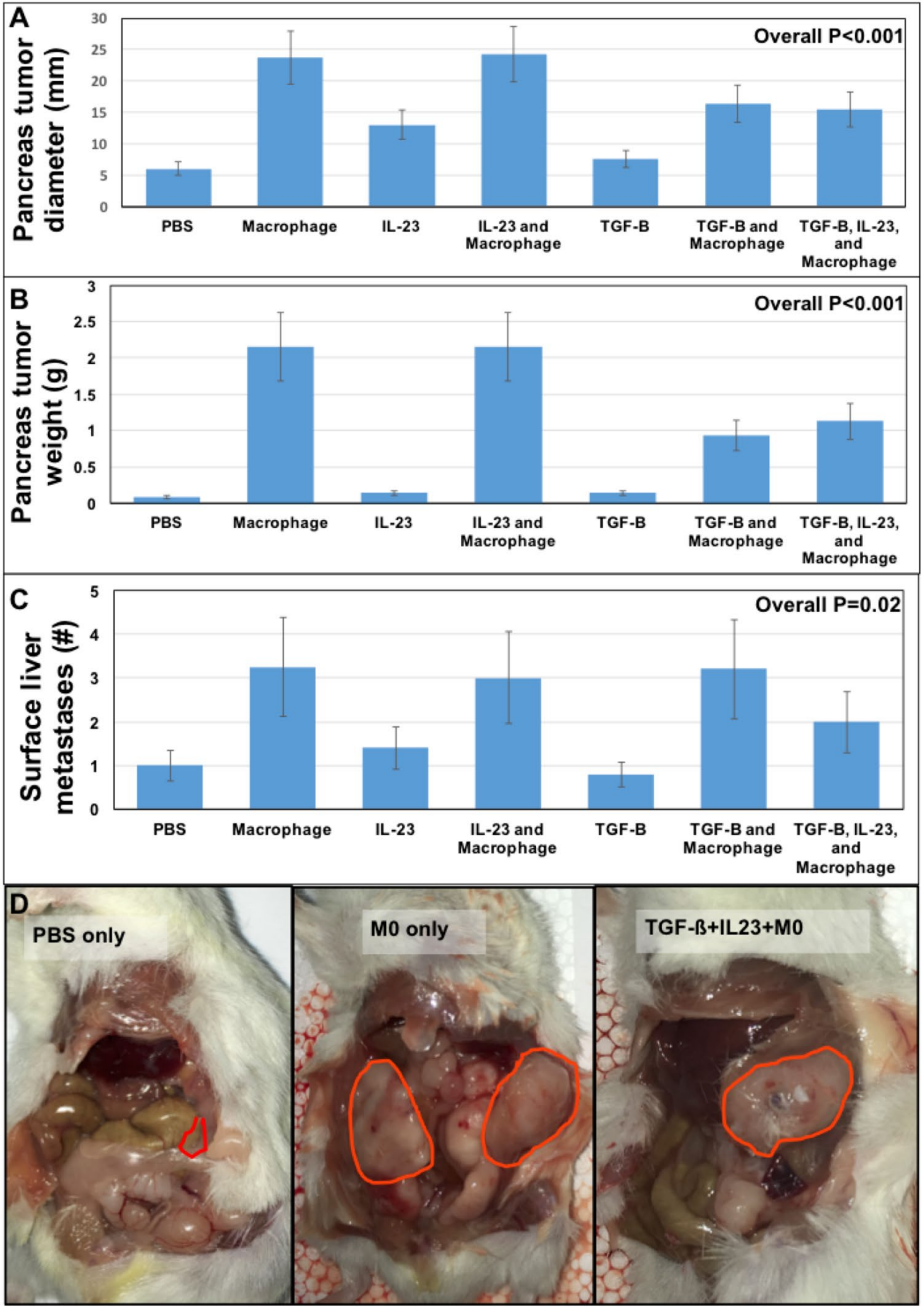
Macrophage co-culture with PDAC *in vivo* increases tumorigenesis and metastases while IL23 and TGF-ß abrogate these phenomena. Panc-1 cells co-cultured with macrophages alone or in combination with IL23 or TGF-ß had primary tumors that were significantly larger by weight and diameter (**A**,**B**, P < 0.001) when Panc-1 cells were exposed to macrophages but this was diminished when pre-treatment included IL23 or TGF-ß. Metastatic PDAC tumors to the liver after orthotopic implantation were larger when co-cultured with macrophages compared to controls (**C**, P = 0.02). Tumor burden after 4 weeks was significantly less when Panc-1 cells co-cultured with macrophages were pre-treated with IL23 and TGF-ß compared to macrophage co-culture alone (**D**). Macrophage co-culture with Panc-1 cells increases tumorigenesis and metastases formation (outlined in red) while combined IL23 and TGF-ß treatment diminishes these phenomena.

In order to understand the importance of macrophages in the pancreatic TME, we co-cultured M2 polarized macrophages (ATCC 9855) 1:10 with Panc-1 cells *in vitro*. We then implanted Panc-1 cells orthotopically into NSG mice (NOD-scid IL2Rγ^null^ with defective/dysfunctional B, T, NK, and macrophage cells) and measured tumor growth after 4 weeks. We found that all groups containing macrophages had significantly higher tumor burden (Fig. 4, P < 0.03) when compared to PBS control treated Panc-1 cells. Treatment with TGF-ß1 or IL23 alone did not modulate primary tumor growth significantly when compared to PBS pre-treatment alone (Fig. 4A,B).

In order to understand the importance of macrophages and TGF-ß in PDAC primary tumor growth, we utilized an orthotopic PDAC model utilizing NSG mice (NOD-scid IL2Rγ^null^). The development of liver metastasis, the most common site of PDAC metastasis, was highest when Panc-1 cells were pre-treated with macrophages and implanted into the pancreas of the mice (Fig. 4C, P = 0.02). Pre-treating cancer cells with TGF-ß1 or IL23 alone demonstrated a similar rate of metastasis when compared to PBS control treated cells. Importantly, by adding IL23 pre-treatment to macrophage/TGF-ß treatment of Panc-1 cells, we found that the rate of liver metastasis decreased to closer to the baseline rate as observed in PBS treated Panc-1 cells (Fig. 4C).

We found a similar effect on the primary PDAC weight and tumor size in this mouse model. By adding IL23 treatment to the Panc-1 cells treated with macrophage + TGF-ß, we found that the weight of primary tumors was less (Fig. 4B, P < 0.001) and PDAC tumor diameter was smaller (Fig. 4A, P < 0.001). In both instances, macrophages were associated with increased Panc-1 tumor growth. Isolated treatments of IL23 or TGF-ß treatments alone were not associated with Panc-1 primary tumor growth *in vivo* but the combined treatments seem to inhibit the aggressive phenotype previously associated with macrophage exposure (Fig. 4D). Macrophage co-cultured with Panc-1 cells increased primary tumor growth and metastases formation while combined IL23 and TGF-ß pre-treatment diminished these phenomena.

## Discussion

The importance of TGF-ß has been well documented in the context of the pancreatic TME as well as the paradoxical nature of its role in PDAC progression^3,32,33^. The relationship between TGF-ß and the immune system has been studied, but primarily these investigations were in the context of downregulation and inactivation of tumor infiltrating T-cells by paracrine TGF-ß signling^10,34^. Our work builds on these studies by incorporating IL23 into a model of the pancreatic TME. Herein, we demonstrate that IL23 signaling contributes an important role in cross talk between PDAC cells and macrophages. While all patients in this study had early stage PDAC, tumors highly expressing IL23 were associated with long-term survival in PDAC. Further investigation noted that a statistically significant association between IL23 and TGF-ß was primarily found in short-term survivors suggesting an association in aggressive PDAC, possibly due to changes secondary to high TGF-ß expression.

We also demonstrated that increased primary tumor growth associated with macrophage exposure in a mouse model was partially abrogated by IL23 and TGF-ß. Specifically, IL23 and TGF-ß pre-treatment diminishes Panc-1 primary tumor growth associated with macrophage co-culture. We also demonstrate in our mouse model that TGF-ß diminishes the metastatic potential of pancreatic cancer while IL23 may enhance this effect (Fig. 4C). We theorize that further work is needed to understand the clinical ramifications of anti-TGF-ß based therapy in PDAC based on the degree of inflammation in the TME. This data suggests that some patients will be harmed by anti-TGF-ß based therapy based on the peri-tumoral interleukin pattern.

IL23, a pro-inflammatory interleukin associated with inflammatory bowel disease and autoimmune diseases^35^, has also been reported to lead to the development of gastrointestinal malignancies^21,26,36^. For example, in Chan *et al*.’s work^26^, pre-malignant duodenal adenomas in mice are associated with IL23 over expression. Others have shown that IL23 induces vascular inflammation potentially allowing for cancer cells to more easily metastasize^36^. In addition, higher tumoral levels of IL23 have been associated with worse prognosis when analyzed on a stage by stage fusion in colorectal cancer patients^21^.

Other studies have questioned the importance and role of IL23 in cancer tumor biology. For example, recent work has suggested that IL23 expression from TAMs is secondary to macrophage M1 polarization and not a relevant feature of the cancer cell per se^5^. Interestingly, small molecule inhibition of TGF-ß receptors coupled with IL23 treatment resulted in significantly decreased head and neck squamous cell carcinoma progression in a mouse model, primarily through lack of tumoriogenesis^27^. Those authors theorized that systemic production of Th17 T cells was the immunologic mechanism by which tumor growth was inhibited.

Tumor associated macrophages create an environment that encourages a more aggressive PDAC cell phenotype characterized by an EMT phenotype, larger primary tumors, and increased rates of metastasis as described herein. We identified that IL23 is in fact expressed by macrophages and Panc-1 cells (Fig. 3), suggesting a role for immune modulation by PDAC cells as part of carcinogenesis. While primarily produced by macrophages, IL23 is produced by other cells such as endothelial cells^5,36^, so it is not completely surprising that IL23 is produced by PDAC cells as well. The “immunoactivity” of PDAC may be one reason that immune therapy against PDAC has not been met with the same success as in other solid tumors such as melanoma^34,37^.

IL23 mediated tumor progression may be context-dependent similar to the effects of TGF-ß being context-dependent. In one setting (duodenal/colon carcinoma), IL23 seems to lead to tumorigenesis through pre-malignant lesions that develop into carcinoma in an inflammatory mediated manner. In another setting (head and neck carcinoma), IL23 is not associated with tumorigenesis. In the head and neck carcinoma mouse model, anti-TGF-ß treatment coupled with IL23 treatment inhibited tumorigenesis^27^. Our data suggests that IL23 has a small role inhibiting PDAC metastases and larger role inhibiting primary tumor growth similar to the role of other interleukins in other maliganncies^38–40^.

In order to investigate the seemingly contrary reports, we investigated both primary PDAC tumor growth and metastasis in a murine model of PDAC with mixed cell orthotopic pancreas implants. It appears that macrophages induce liver metastasis and primary tumor progression in orthotopically implanted PDAC whereas IL23 tempers liver metastasis development by about 25% (Fig. 4C, P = 0.02). Further investigation yields that IL23 has minimal effects on primary PDAC weight but TGF-ß decreases tumor weight by nearly 50% (Fig. 4B, P < 0.001) and decreases tumor diameter by 25% (Fig. 4A, P < 0.001). It is concerning that this data would suggest that some patients will be harmed by anti-TGF-ß based therapy.

This study has several limitations that must be noted. First, there are many other signaling molecules between macrophages and PDAC cells. In these studies, we pre-polarized macrophages towards type 2 macrophages, more similar to true tumor associated macrophages^11,41^. However, the polarization of macrophages is a spectrum, and it is likely that Panc-1 cells, likely via endogenous TGF-ß, modulates the co-culture macrophage polarization. Second, while we demonstrate that macrophages are associated with tumor progression in Panc-1 cells, it is possible that more advanced PDAC models (i.e., cell lines derived from metastatic deposits) may react differently to macrophages exposure or anti-TGF-ß therapy. Furthermore, our approach to this was to pre-treat cells prior to implantation. It is possible that ongoing exposure may change our results. The risk of this, however, is that IL23 or TGF-ß may artificially effect the mouse (host) of this model. A more refined patient derived model, such as pancreatic organoids, will improve future investigations. The lack of mechanistic data limits the utility of this research but provides a path for future investigations. Our results are also suggestive and congruent with other studies^11–14^ that numerous other interleukins, such as IL10, play a role in modulating the macrophage-PDAC cell interaction, but the complete set of interleukins remains to be elucidated. The lack of an innate immune systems in this mouse model is a limitation that will be addressed through future syngeneic mouse models with spontaneous PDAC tumors (i.e., KPC), patient derived orthotopic organoids, and patient derived orthotopic implants.

Macrophages serve a critical role in PDAC tumor progression and metastasis. TGF-ß, a known tumor suppressor in early PDAC, may exert a role in creating a less tumorigenic TME through regulation of macrophages. Future work is needed to understand the mechanism of this cross talk and the ultimate role served by IL23 in modulating tumor associated macrophages. As anti-TGF-ß based therapies enter clinical practice, their mechanisms of action and consequences must be fully understood in order to avoid the negative effects of anti-TGF-ß in the pancreatic immune TME.

## **Patients**, **Materials**, **and Methods**

### Human subjects

This study was approved by the institutional review board at both institutions (Washington University in St. Louis Institutional Review Board and the University of Tennessee Health Science Center Institutional Review Board). All experiments were performed in accordance with relevant guidelines and regulations.

24 long term survivors (>30 months) and 24 short term survivors (<12 months) with resected PDAC were identified from the Washington University in St. Louis Pancreatic SPORE Tumor Registry. Tumor sections were taken from formalin fixed, paraffin embedded blocks and protein expression of IL23 and TGF-ß was independently investigated with immunohistochemistry utilizing quantitative analysis with CellProfiler image analysis software. Immunohistochemistry expression (quantitative DAB colorimetric analysis) of IL23 or TGF-ß was determined to be high (highest quartile), low (lowest quartile), or median (within the interquartile range) expression based on 5 representative images of each tumor section. The quartile ranges were based on quantitative protein expression analysis of all tumor specimens combined (both long- and short-term groups combine to define the 25^th^ and 75^th^ percentiles defining the quartiles of protein expression for each protein).

In order to prepare the slides for analysis, slides were de-paraffinized and rehydrated with heat, xylene bath, and sequential ethanol washes. Antigen retrieval was performed with a heated citrate buffer solution. The primary antibodies against IL23 (ThermoFisher Scientific, PA5-20239) and TGF-ß1 (Novus, NBP1-67698) were both diluted 50:1. To the slides, HRP conjugated secondary antibody was then added, washed, and finally, DAB-substrate (brown) was added for 5 mins. This was then washed off. Counter stain was performed with hematoxylin (blue) and cover slips were applied.

### The Cancer Genome Atlas (TCGA) analysis

Gene expression data was obtained from The Cancer Genome Atlas (TCGA) and analyzed as previously described^17^. Briefly, IL23 mRNA gene expression was quantified amongst all patients samples with PDAC in the TCGA. Groups were established as lowest quartile, highest quartile, and the interquartile range. We then analyzed the same PDAC samples to determine whether or not an individual tumor specimen expressed TGF-ß1 mRNA in the top quartile of all samples (“high expressing”). The percent of “high expressing” TGF-ß1 mRNA expressing tumors in each of the IL23 mRNA gene expression groups was calculated.

### Cell lines and cell culture

The human PDAC cell line, Panc-1 (ATCC CRL-1469), and the human macrophage cell line (ATCC, CRL 9855) were obtained from the American Type Cell Collection (Manassas, VA) and kept in the recommended complete growth media with fetal bovine serum and 1% penicillin/streptomycin. Cells were subculture when they reached 90% confluence and all experiments were performed with passages less than 30.

### Orthotopic xenograft tumor model

Panc-1 cells were pre-treated with PBS (control), IL23 (10 ng/mL, recombinant BioLegend), TGF-ß1 (10 ng/mL, recombinant, BioLegend), macrophage exposure (10:1 ratio of Panc-1 cells to macrophages), or combinations of these treatments. Macrophages were co-cultured 1 week with Panc-1 cells. IL23 and TGF-ß1 treatments were for 24 hours at a time, thrice weekly for 1 week.

After that, 1 million Panc-1 cells were implanted in the pancreatic tail of each NSG mouse (NOD-scid IL2Rγ^null^ with defective/dysfunctional B, T, NK, and macrophage cells). Briefly, mice underwent anesthesia according to our approved protocol (University of Tennessee Health Science Center Institutional Animal Care and Use Committee). All experiments were performed in accordance with relevant guidelines and regulations.

Fur was removed from the abdomen and it was sterilely prepped. Using sterile technique, a small incision was made on the upper left abdomen. The spleen and stomach were identified and the pancreatic tail was raised out of the abdomen. 5 mice in each group had 1 million Panc-1 cells in Matrigel (cell suspension in 1:1 ratio DMEM:Matrigel by volume in a total volume of 50 μL per mouse) were injected into the pancreatic body/tail. The pancreas and spleen were placed back into their anatomic position and the abdominal muscle/peritoneal lining was close with absorbable suture. The skin was closed with clips. Mice were then placed back into their cage. Clips were removed 10 days later. Mice were euthanized 4 weeks later.

Upon sacrificing the mice, the pancreas and liver were removed. The pancreas tumor weight and diameter were recorded after separating it from the pancreas. The liver was removed and the number of metastases on the surface of the liver were counted.

### SDS-PAGE and Western Blot

Briefly, human macrophage cell line (ATCC, CRL 9855) was treated with Phosphate-Buffered Saline (PBS, control) and Interleukin-23 (Biolegend). The cells were harvested at various time points, washed once with PBS, lysed with RIPA buffer containing 1× protease and phosphatase inhibitors, protein samples quantitated (Quick Start Bradford Dyed Reagent, Biorad), and 100 micrograms each of protein samples were analyzed by SDS-PAGE (10% gel) followed by Western blotting, and autoradiography. Precision Plus Dual Color (Biorad) was used as protein standard marker. TGFβ1 antibody (Novus, rabbit polyclonal), IL23 p19 antibody (ThermoFisher, rabbit polyclonal) β-actin antibody (Cell Signaling Technology, mouse mAb), anti-rabbit-HRP and anti-mouse-HRP (Cell Signaling Technology) were all used at 1:1000 dilution by following manufacturer’s instructions. Clarity Western ECL Substrate (Biorad) was used to develop the probe signal, and Classic Blue BX film (MidSci) was used for autoradiography.

### Statistical analysis

STATA version 14 (StataCorp, College Station, TX) and Excel (Microsoft Corp, Redmond, WA) was used for data analysis and graph creation. Uncertainties represent standard deviations. Statistical significance, α, was set to P = 0.05. For quantitative IHC analysis of resected specimens, comparisons with clinical outcomes were investigated with Student’s t-test, ANOVA, and multivariate regression.

## Electronic supplementary material

Supplementary Info
