# Supplementary material for: IL23 and TGF-ß diminish macrophage associated metastasis in pancreatic carcinoma
Source: Sci Rep. 2018 Apr 11;8:5808. doi: 10.1038/s41598-018-24194-5 (PMC5895618; doi:10.1038/s41598-018-24194-5)

**­­IL23 and TGF-ß diminish macrophage associated metastasis in pancreatic carcinoma**

S. Mazher Hussain^1^, Leighton F. Reed^1^, Bradley A. Krasnick^2^, Gustavo Miranda-Carboni^1^, Ryan C. Fields^2^, Ye Bi^2^, Abul Elahi^1^, Abidemi Ajidahun^1^, Paxton V. Dickson^1,3^, Jeremiah L. Deneve^1,3^, William G. Hawkins^2^, David Shibata^1,3^, Evan S. Glazer^1,3^

^1^University of Tennessee Health Science Center, Memphis, TN

^2^Barnes-Jewish Hospital and The Alvin J. Siteman Cancer Center, Washington University in St. Louis, St. Louis, MO

^3^UT West Cancer Center, Memphis, TN

**Supplementary Information**

**Figure 3 original blots**

TGF-β protein expression after IL23 treatment in macrophages


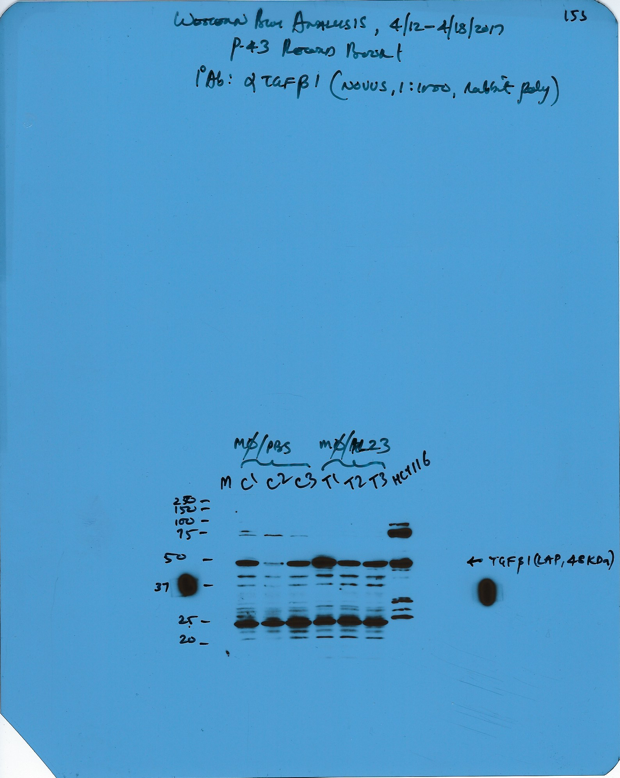


β-actin protein expression after IL23 treatment in macrophages


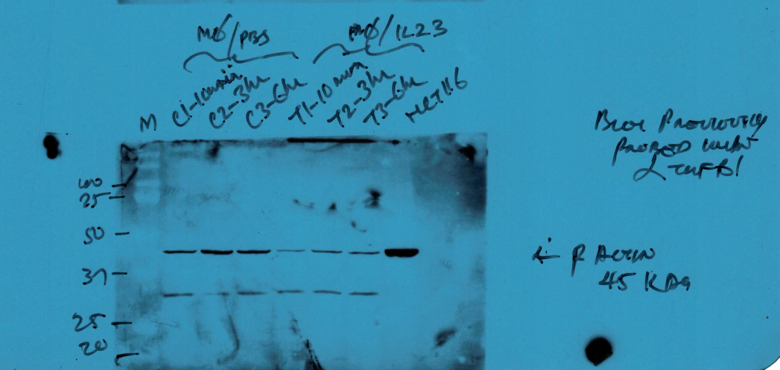


Pan-NOS (nNOS, eNOS, or iNOS) expression was not significantly altered upon macrophage treatment with IL23 compared to PBS controls. Non-specific NOS breakdown products and low expression at the expected molecular weight was identified as marked.


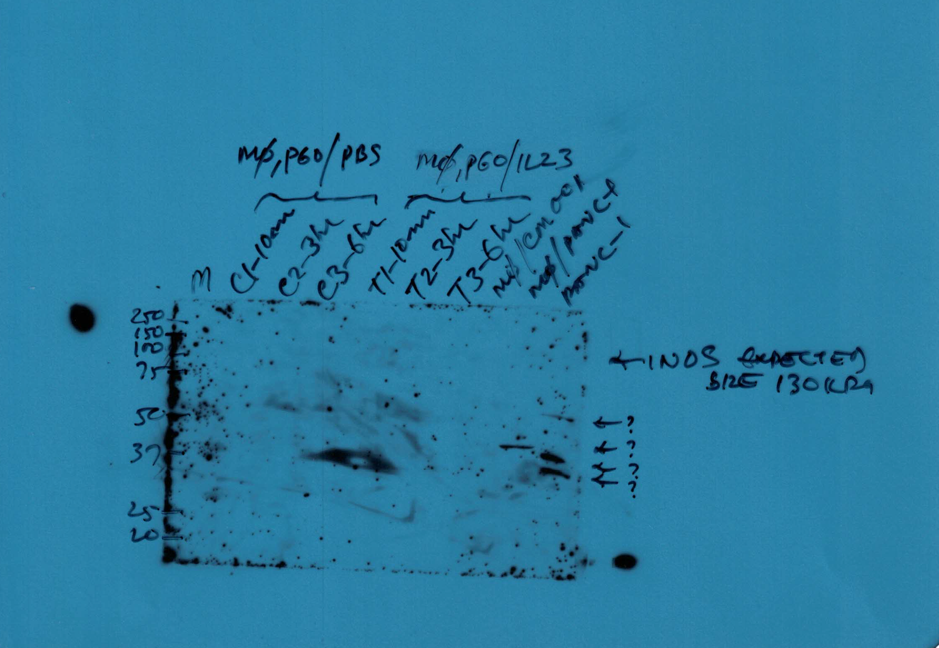

Supplement: Supplementary file 1 — Supplementary Info [file 41598_2018_24194_MOESM1_ESM.docx]
